# Management and Treatment of Patients With Major Depressive Disorder and Chronic Diseases: A Multidisciplinary Approach

**DOI:** 10.3389/fpsyg.2020.542444

**Published:** 2020-09-24

**Authors:** Susana Sousa Almeida, Francesca Benedetta Zizzi, Agnese Cattaneo, Alessandro Comandini, Giorgio Di Dato, Ennio Lubrano, Clelia Pellicano, Vincenza Spallone, Serena Tongiani, Riccardo Torta

**Affiliations:** ^1^Portuguese Institute of Oncology Porto (IPO Porto), Hospital Cuf Porto (HCuf Porto), University of Porto (FMUP), Porto, Portugal; ^2^Neurology, Psychiatry and Psychology Unit, Koelliker Hospital, Turin, Italy; ^3^Angelini RR&D (Research, Regulatory & Development) – Angelini S.p.A, Rome, Italy; ^4^Academic Rheumatology Unit, Dipartimento di Medicina e Scienze della salute “Vincenzo Tiberio”, Università degli Studi del Molise, Campobasso, Italy; ^5^Laboratory of Neuropsychiatry, Istituto di Ricovero e Cura a Carattere Scientifico (IRCCS) Santa Lucia Foundation, Rome, Italy; ^6^Division of Endocrinology, Department of Systems Medicine, University of Rome Tor Vergata, Rome, Italy; ^7^Clinical Psychology and Psycho-Oncology Unit, Department of Neuroscience “Rita Levi Montalcini”, University of Turin, A.O.U. “Città della Salute e della Scienza” Hospital, Turin, Italy

**Keywords:** depression, major depressive disorder, chronic diseases, diabetes, rheumatoid arthritis, cancer, Parkinson’s disease

## Abstract

In patients with physical chronic diseases, the prevalence of major depressive disorder (MDD) is approximately 2‐ to 3-fold higher than in the general population, and it can reach up to 20–40%. The comorbidity of MDD with chronic medical diseases is associated with poorer quality of life, increased medical symptom burden, poor adherence to self-care regimens, increased risk of functional impairment, morbidity, and mortality, and also higher medical costs. Despite this evidence, in routine practice, psychological issues and concerns are frequently inadequately managed. This consensus document proposes that a proper diagnosis, a multidisciplinary approach, and a personalized treatment plan would allow patients with MDD and chronic comorbidities to be more compliant, to improve the outcomes, to reduce possible relapses in the long term, and to prevent or better manage complications and adverse events. This proposal might be useful for any health professionals who deal with patients with chronic diseases, as it can help to pay more attention to the emotional impact of these conditions, in particular in terms of depressive symptoms.

## Introduction

The prevalence of major depressive disorder (MDD) in the general population is approximately 6.6% (Kessler et al., 2005), with a lifetime prevalence of approximately 11% (Lim et al., 2018). In patients with chronic diseases, MDD prevalence is approximately 2‐ to 3-fold higher, notably reaching up to 20–40% (Read et al., 2017). A higher prevalence of MDD has been found in patients with a range of chronic conditions, including cardiovascular diseases (Rudisch and Nemeroff, 2003), diabetes (Anderson et al., 2001), arthritis (Matcham et al., 2013), and cancer (Smith, 2015). The WHO World Health Survey (WHS) found a greater prevalence of MDD in people who had at least one chronic condition (9.3–23%), compared to those with none (3.2%; Moussavi et al., 2007). The comorbidity of MDD with chronic diseases has been associated with poorer quality of life, increased symptom burden, poor adherence to self-care regimens, increased risk of morbidity and mortality, and higher medical costs (Moussavi et al., 2007; Katon, 2011; Reddy, 2016). Beyond that, functional impairment is more severe in patients with comorbidity compared to that in patients with either MDD or a chronic disease exclusively (Moussavi et al., 2007; Kang et al., 2015).

Despite this, clinicians frequently fail to address the psychological component of chronic diseases. Depressive symptoms are often inadequately managed because of a lack of training, guidelines, and recommendations (Read et al., 2017). In a fundamental attempt to fill this gap, this multidisciplinary consensus document was produced with two main purposes: (1) to describe the prevalence of MDD in patients with chronic diseases (i.e., diabetes, rheumatoid arthritis, cancer, and Parkinson’s disease) and (2) to define guidelines to manage this specific patient population. The multidisciplinary international advisory board was composed of European experts with proven experience in caring for patients with chronic diseases and MDD. The expert consensus statement rests on literature evidence. An extensive review of the literature was performed to identify studies investigating prevalence, clinical manifestation, and impact on outcome of MDD in patients with chronic diseases.

## Materials and Methods

The review was conducted according to the Preferred Reporting Items for Systematic Review and Meta-Analyses (PRISMA) guidelines (Moher et al., 2009). Four electronic databases (MEDLINE, PubMed, CINAHL, and PsychINFO) were searched to identify studies. The following terms were used in combination with a range of subject headings for each database: (depression OR depressive OR mdd OR mood OR psychiatr^*^ OR psychol^*^) AND (diabetes OR diabet^*^ OR rheumatoid arthritis OR arthritis OR rheumat^*^ OR cancer OR tumor OR oncol^*^ OR Parkinson’s disease OR Parkinson OR chronic illness OR progressive disease).

### MDD and Chronic Diseases: A Close Relationship

The relationship between MDD and chronic diseases is bidirectional (Katon, 2011). Physical symptoms can cause or exacerbate depressive symptoms, but the reverse also occurs, with depressive symptoms antedating the onset of health problems (Katon, 2003). The adverse health risk behaviors and the psychobiological changes associated with MDD can increase the risk for chronic diseases, and the biological changes and complications associated with chronic medical conditions can precipitate depressive symptoms leading to MDD (Katon, 2011). Chronic physical morbidity can also lead to MDD through psychosocial factors such as symptom burden, disability, decreased quality of life (Katon, 2003), pain (Bair et al., 2003), dysfunctional beliefs about disease, and ineffective coping (Ziarko et al., 2014). MDD can worsen the outcomes of chronic diseases because of its effect on proinflammatory factors, the hypothalamic-pituitary axis, the autonomic nervous system, and metabolic factors, in addition to being associated with a higher risk of engaging in health risk behaviors (Katon, 2011). Furthermore, poorer disease management may occur in MDD patients, as they less likely adhere to medical regimens (Alexopoulos et al., 2008; Katon, 2011). Although a cause-effect relationship between MDD and chronic diseases has not been established, there is growing evidence that the neurobiological changes of chronic diseases and the associated psychological distress can lead to MDD (Read et al., 2017). Vascular brain lesions, reductions of neurotransmitters in the limbic area, hyperactivity of the hypothalamic-pituitary-adrenal axis, hormonal, metabolic, and immune-inflammatory dysregulations are common in many chronic diseases, and have been linked also to MDD (Nestler et al., 2002; Camus et al., 2004; Freeman et al., 2004; Pariante and Lightman, 2008; Bogdan et al., 2013; Miller and Raison, 2016; Penninx, 2017). A persistent inflammatory status has been frequently found in the course of many chronic diseases and has been associated with a higher risk for MDD (Nestler et al., 2002; Miller and Raison, 2016). As MDD has been linked to low-grade inflammation/dysregulated inflammation, in the long term such condition may predispose to the onset of a chronic disease (Miller and Raison, 2016).

### MDD and Diabetes

Patients with MDD and those with diabetes patients share reciprocal susceptibility and a high degree of comorbidity (Canadian Diabetes Association Clinical Practice Guidelines Expert Committee et al., 2013; Pouwer, 2017). The prevalence of depressive symptoms among patients with diabetes is in the range of 30%, and the prevalence of MDD is approximately 10%, which is double the overall prevalence in people without a chronic disease (Canadian Diabetes Association Clinical Practice Guidelines Expert Committee et al., 2013). Risk factors for MDD in patients with diabetes have been identified as female sex, adolescents/young adults and older adults, poverty, few social supports, stressful life events, poor glycaemic control, longer duration of diabetes, and presence of long-term complications, especially painful neuropathy (Mezuk et al., 2008; D’Amato et al., 2016). In addition, the complexity of diabetes treatment regimens might be highly demanding and its burden can lead to depressive reactions. MDD patients have an approximately 60% increased risk of developing type 2 diabetes (Mezuk et al., 2008). The possible mechanisms leading to diabetes in MDD patients involve adverse health risk behaviors (such as physical inactivity and obesity, responsible for insulin resistance) and psychological distress, which causes chronic hypothalamic-pituitary-adrenal activation with subclinical hypercortisolism (Mezuk et al., 2008; Joseph and Golden, 2017). Moreover, long-term use of antidepressants has been related with an increased risk of diabetes (Andersohn et al., 2009). In patients with type 1 diabetes, the presence of MDD has been associated with severe hyperglycaemia and hypoglycaemia (Gilsanz et al., 2018) and with increased cardiovascular mortality (Pouwer, 2017; Farooqi et al., 2019). The prognosis for comorbid MDD and diabetes is worse than when each illness occurs separately: MDD amplifies the diabetes symptom burden and worsens clinical outcomes. On the other hand, MDD tends to be longer and have a higher chance of recurrence (Peyrot and Rubin, 1999; Ludman et al., 2004; Egede et al., 2005).

### MDD and Rheumatoid Arthritis

The prevalence of MDD can be as high as 66% in patients with rheumatoid arthritis and almost 17% of rheumatoid arthritis patients have a current MDD (Matcham et al., 2016; Fiest et al., 2017). Chronic pain and depressive symptoms are closely related as regards occurrence, and the coexistence tends to further aggravate the severity of both conditions (Sheng et al., 2017). Although the nature of the relation between pain and MDD remains unclear, recent studies have found considerable overlaps between pain‐ and depression-induced neuroplasticity and neurobiological changes (Sheng et al., 2017). Many studies have described the associations between peripheral and brain immune responses, which suggest shared pathophysiological mechanisms for immune-mediated inflammatory diseases and MDD (Nerurkar et al., 2019). MDD is associated with adverse outcomes in rheumatoid arthritis: higher levels of pain and disability, lower quality of life, increased disease activity, reduced response to treatment, decreased likelihood of achieving symptom remission, and increased mortality (van den Hoek et al., 2016; Fiest et al., 2017).

### MDD and Cancer

The rate of MDD in cancer patients is thought to be up to three times higher than that in the general population (Linden et al., 2012). Depressive symptoms of cancer patients exist on a continuum ranging from non-pathological sadness to an adjustment disorder to subclinical depression to MDD: it has been reported that 0–38% of cancer patients have a concomitant MDD and 0–58% suffer with depressive symptoms (Smith, 2015; Sherrill et al., 2017). MDD rates may vary depending on the site of primary cancer, age, and sex (Linden et al., 2012) and differ over the course of cancer. High rates of MDD are frequent around the time of diagnosis and in patients with advanced disease, while in cancer survivors 5 years after diagnosis, they are comparable to those of the general population (Linden et al., 2012; Smith, 2015). Metastases and pain have been associated with more severe MDD (Ciaramella and Poli, 2001) and the prevalence of MDD is significantly higher in patients with pain, suggesting how pain may have a causative role in MDD onset (Spiegel et al., 1994). MDD in cancer is a multifactorial disorder involving psychosocial (psychological distress, maladaptive coping, previous mental disorders, and social and emotional support), biological (inflammation, hypothalamic-pituitary-adrenal axis activation, neurotransmitters, and hormones changes), and iatrogenic mechanisms (antiemetic drugs and immunotherapy agents, including INF-α; Leonard, 2010; Smith, 2015). MDD in cancer leads to a poorer quality of life, compromises cancer outcomes, and results in higher rates of mortality (Satin et al., 2009; Smith, 2015).

### MDD and Parkinson’s Disease

The prevalence of MDD in Parkinson’s disease (PD) varies between studies (Aarsland et al., 2011). However, a systematic review estimated that 50–70% of PD patients have been affected by MDD (Bomasang-Layno et al., 2015). MDD in PD is more common than in age-matched controls with other chronic diseases, such as diabetes, hypertension, coronary artery disease, or congestive heart failure (Nuti et al., 2004; Egede, 2007; Reijnders et al., 2008; Tan, 2012) and can develop at any phase of the disease. Studies suggested that depressive symptoms may precede the diagnosis of PD by 5 up to 20 years, and MDD has been associated with an increased risk of developing PD with an incidence of 23% (Chaudhuri et al., 2006; Pellicano et al., 2007; Postuma et al., 2012; Chen et al., 2013; Pont-Sunyer et al., 2015). Once PD is diagnosed, the annual rates of newly diagnosed MDD range from 1.86 to 10% (Aarsland et al., 2011; Marsh, 2013; Yapici Eser et al., 2017) and studies have shown that MDD tends to be persistent and worsens over time (Aarsland et al., 2011). Although the exact etiology is unknown, several hypotheses supporting the link between MDD and PD physiopathology have been proposed (Aarsland et al., 2011; Santiago et al., 2017). A significant biological mechanism is more probable than a pure reactive basis and could be a result of damage to serotoninergic neurotransmission, as well as limbic noradrenergic and dopaminergic mechanisms (Chaudhuri et al., 2006). Other factors that might contribute to MDD in PD patients are stress-induced hypercortisolaemia, pain, genetic abnormalities, inflammation and changes in neurotrophic molecules, previous mood disorders, and psychosocial distress (Leonard, 2010; Aarsland et al., 2011; Santiago et al., 2017). In PD, MDD is associated with worse clinical outcomes: when untreated, MDD is related to earlier initiation of dopaminergic therapy, greater functional disability, faster physical and cognitive deterioration, increased mortality, poorer quality of life, and increased distress in caregivers (Aarsland et al., 2011; Marsh, 2013).

### MDD in Chronic Diseases: Diagnostic Problems

Although MDD is frequent in chronic disease patients, it remains often unrecognized and, despite the negative consequences on patient health, physicians tend to undertreat it (De Jean et al., 2013). One reason for this could be the diagnostic difficulty experienced by other specialists rather than psychiatrists. MDD may be underdiagnosed when comorbid with a chronic disease, as it may be disguised by other symptoms that draw the attention of both patient and physician. Somatic disturbances such as fatigue, appetite disturbances, and sleep disorders may be sequelae of physical problems rather than symptoms of MDD. In addition, the presence of pain could further complicate the diagnosis. To discern these situations, tools that highlight emotional, cognitive, and somatic aspects of pain are useful; for instance, symptoms such as hypo-volition and anhedonia need to be taken into consideration during anamnesis to establish the presence of depressive symptoms, regardless of pain. Additionally, patients with chronic diseases may deny MDD, whereas clinicians may sometimes have insufficient time to investigate the psychological component. Moreover, the stigmatization of MDD by both physicians and patients may jeopardize diagnoses. Furthermore, MDD is a complex combination of physical and mental symptoms, and when organic factors are involved, as in chronic diseases, patients struggle to accept treatment for psychological comorbidity (De Jean et al., 2013). General practitioners represent the first reference for patients with chronic diseases. However, they seem often unaware that MDD is common among them and that MDD affects the course of the diseases and clinical outcomes. For these reasons, recommendations for MDD diagnostic algorithms and screening practices in chronic diseases are needed.

### Management of MDD in Chronic Diseases: The Multidisciplinary Team Approach

The National Institute for Health and Care Excellence (NICE) recommendations state that patients with chronic diseases and moderate to severe MDD, linked with functional impairment, should be treated with a multidisciplinary team approach [National Collaborating Centre for Mental Health (UK), 2010; Kang et al., 2015]. Collaborative care should be part of a stepped-care program and coordinated at the primary and secondary care level; all sectors of care should be integrated in a comprehensive approach to both mental and physical symptoms. A multidisciplinary team (including general practitioners, nurses, specialists, and mental health professionals) coordinated by a case manager should closely collaborate to provide a wide range of interventions [e.g., patient education, psychological and pharmacological interventions, medication management, follow-up controls; National Collaborating Centre for Mental Health (UK), 2010]. This approach has been shown to be more effective than usual care as regards illness burden, physical, and psychological outcomes in patients with MDD and chronic diseases (van Eck van der Sluijs et al., 2018). The first step to manage MDD in chronic diseases patients is the communication of the diagnosis as a step of the whole care process. Physicians should help to understand the complexity of the disease, focusing on the mutual relationship between organic, emotional, and cognitive aspects. A more structured physician-patient relationship has been associated to better outcomes (Linzer et al., 2015). Moreover, well-informed patients tend to be more autonomous and adherent to treatment. A psychological evaluation should be implemented to detect or prevent psychological morbidity. A short preliminary assessment could be performed using rapid screening tools, such as the Hospital Anxiety and Depression Scale (an instrument developed to detect symptoms of anxiety and depression in patient with medical diseases) and the Distress Thermometer [Vodermaier et al., 2009; National Collaborating Centre for Mental Health (UK), 2010]. In case of positive screening, physicians must deepen the assessment and consider a psychological or psychopharmacological intervention, or, when symptoms are more severe, refer the patient to the psychiatrist or clinical psychologist. Educational programs may increase their sensitivity to MDD manifestations in chronic diseases and their comfort in the choice of treatment options. Table 1 provides a detailed summary of some key-components to implement and sustain a multidisciplinary team approach to manage MDD in chronic diseases.

**Table 1:** Key-components to implement and sustain a multidisciplinary team approach to manage major depressive disorder (MDD) in chronic diseases.

Component	Features and methods
Developing a shared individualized care plan	• Collecting a patient’s biopsychosocial semi-structured history of MDD and chronic illness (diagnoses, treatments, and complications) from different perspectives (psychiatric, medical, psychological, and nursing)
	• Understanding and sharing the patient’s explanatory model of the disease
	• Negotiating the therapeutic alliance and identifying goals together with the patient from each perspective
	• Sharing decision making processes within the team
	• Explaining all the treatments to the patient in phases as an integrated process
	• Sharing the information and relevant events within the team and defining a case manager responsible for keeping the team and the patient updated
Systematic monitoring of the care plan	• Tracking the patient’s relevant clinical data in an electronic medical record accessible to the team members
	• Discussing progress, caseload, and resistance encountered by the patient in team supervisions
	• Assessing the patient’s needs and identifying new professionals, roles, and resources outside of the team required to meet them
Support of the patient self-care	• Providing tailored educational materials from each perspective to the patient
	• Fostering the patient’s motivation to get better as a shared message
	• Monitoring and promoting the patient’s adherence to the treatments and prescriptions mutually
Team training	• Organizing face-to-face training sessions to consolidate interprofessional collaboration ties and to develop a common language within the team
	• Encouraging the expression of needs, doubts, disagreement within the team
	• Planning scheduled training update sessions based on challenges and success

### Management of MDD in Chronic Diseases: The Problem of Patient Adherence

Nearly 50% of patients with chronic diseases fail to adhere to medical directives, regardless of the drug latency or efficacy (Zolnierek and Dimatteo, 2009; Miller, 2016). It is important to pay attention to the psychological status of chronic diseases patients, as MDD has been associated with poor adherence (Moussavi et al., 2007; Katon, 2011; Reddy, 2016). Chronic patients’ adherence may be influenced by many factors: understanding of the disease and treatment, beliefs about the benefits and efficacy of prescribed regimens, side effects, financial constraints, psychological conditions, and social support (Miller, 2016). To improve adherence, providing the patient with clear information about disease and treatment is fundamental to promote motivation to heal and appropriate health behaviors and also to reduce drop-out related to side effects or latency of clinical response (Miller, 2016). The relative importance of some symptoms for physicians, patients, and caregivers may be different, and these differences could contribute to explaining the adherence to some prescriptions and the withdrawal of others. Clinicians should keep this in mind. A multidisciplinary team approach may help even in this case, as the whole therapeutic management can be a real challenge for the single physician. More specifically, the adherence to psychological and psychopharmacological treatments in chronic diseases patients with MDD is a delicate matter. Tolerability and compliance should be assessed on the long-term and the multidisciplinary team approach should start from the first visit, where the psychiatrist/psychologist can help patients to understand the therapeutic role and potential side effects of the therapy, increasing adherence (Freedland et al., 2011). To reduce the high number of prescriptions and complexity of whole treatment of chronic diseases, the implementation of drugs targeting different symptoms may also increase adherence. Antidepressants, for example, that are effective in managing pain and depressive symptoms, may be particularly useful in the treatment of patients with diabetes, rheumatoid arthritis, cancer, and Parkinson’s disease, where pain and depressive symptoms are frequently concomitant as stated above (Schreiber et al., 2015; Khouzam, 2016).

## Conclusion

A proper diagnosis, a multidisciplinary team approach, and a personalized treatment plan would allow patients with MDD and chronic diseases to be more compliant, to achieve better results, to reduce possible relapses, and to manage or avoid complications and some adverse events. To provide effective care to patients suffering from chronic diseases, health professionals have to appraise the role of psychosocial factors in the genesis and maintenance of these conditions, while recognizing how emotions and cognitions can influence response to treatment and the course of illness, paying particular attention to the presence of depressive symptoms. Further research on the relationship of MDD and chronic diseases and attention of health policy stakeholders are strongly encouraged.

## Author Contributions

All authors listed have made a substantial, direct and intellectual contribution to the work, and approved it for publication.

### Conflict of Interest

SA, EL, CP, VS, and RT acknowledge a consultant fee for the Advisory Board participation from Angelini Pharma. ACa, ACo, GD, and ST are employed by Angelini Pharma.

The remaining author declares that the research was conducted in the absence of any commercial or financial relationships that could be construed as a potential conflict of interest.
